# Effects of Weir Construction on Phytoplankton Assemblages and Water Quality in a Large River System

**DOI:** 10.3390/ijerph15112348

**Published:** 2018-10-24

**Authors:** Hae-Jin Lee, Hae-Kyung Park, Se-Uk Cheon

**Affiliations:** 1 Nakdong River Environment Research Center, National Institute of Environmental Research, Goryeong 40103, Korea; hjlee76@korea.kr; 2 Geum River Environment Research Center, National Institute of Environmental Research, Okcheon 29027, Korea; sucheon@korea.kr

**Keywords:** weir construction, *Microcystis*, *Stephanodiscus*, total phosphorus, water environment

## Abstract

Flow regulation is one of the most common anthropogenic factors affecting rivers worldwide. In Korea, 16 weirs were constructed along four major rivers from 2009 to 2012. This study aimed to elucidate initial changes in physical, chemical, and biological variables after the construction of consecutive weirs on the Nakdong River, a major large river system. Water quality variables and phytoplankton cell densities were investigated at eight representative sites and compared with the data recorded before the weir construction. There were spatial and temporal changes in the hydraulic retention time (HRT), total phosphorus (TP), and chlorophyll *a* concentrations among the eight weir sections. HRT increased after the weir construction, while TP and chlorophyll *a* tended to decrease from the middle to lower section of the Nakdong River. Furthermore, differences were observed in the phytoplankton community composition between 2006–2007 and 2013. There was a marginal decrease in the duration of centric diatom (*Stephanodiscus hantzschii*) blooms after weir construction. However, *Microcystis aeruginosa* proliferated more extensively during summer and autumn than it did before the weir construction. Our results suggest that changes in hydrological factors, in response to consecutive weir construction, may contribute to greater physical, chemical, and ecological variability.

## 1. Introduction

The ecological effects of hydrological changes, including the water level, river flow, water velocity, and hydraulic retention time (HRT), on aquatic ecosystems have often been studied. Hydrological changes alter the physical, chemical, and biological characteristics of a river [1,2,3,4,5] and cause habitat fragmentation within rivers, as well as changes in nutrient cycling and primary productivity [6]. Phytoplankton is a source of food and energy for organisms at higher trophic levels within an aquatic ecosystem [7,8], and phytoplankton composition usually changes with seasonal fluctuations in water temperature [9]. The phytoplankton species composition and biomass depend, in a complex manner, on various environmental factors, including the concentration of nutrients (nitrogen and phosphorus) derived from watersheds, light, water temperature, flow, turbidity, and HRT [10,11,12,13,14,15,16].

Since the early 1990s, water quality has deteriorated in the Nakdong River, the second largest river in South Korea, resulting in problems of its use as a source of drinking water, especially downstream. Harmful cyanobacterial (*Microcystis aeruginosa*) blooms from summer to autumn (June to September) and small centric diatom (*Stephanodiscus hantzschii*) blooms from late -autumn to next spring (November to April) are mostly responsible for the deterioration in water quality [17,18,19].

However, significant changes in hydrodynamic conditions have occurred recently in the Nakdong River. Eight artificial weirs were constructed over a stretch of approximately 200 km on the mainstream of the Nakdong River from 2009 to 2012. After the weir construction, the water level and storage capacity of the system have increased, affecting not only the physical and chemical environment of the water, including dilution and accumulation of nutrients, but also the structure of phytoplankton communities [1,4,5]. Studies have shown that drastic changes in the hydrodynamic conditions alter water quality variables and the ecosystem [4,20,21]. However, it has not been elucidated how physical changes in a large river system, with consecutive weirs, affect chemical and biological parameters, such as the water quality and phytoplankton distribution.

The present study was based on the following hypotheses: (1) the physical and chemical environmental conditions in the Nakdong River would be affected, both spatially and temporally, by changes in its hydraulics, due to the construction of consecutive weirs, and (2) the phytoplankton community composition and cyanobacterial cell density would vary spatially and temporally, depending on the hydraulics in a weir section. Based on these hypotheses, we compared and analyzed environmental variables and the phytoplankton composition before and after the construction of weirs on the Nakdong River.

## 2. Materials and Methods

### 2.1. Study Area and Sampling Sites

**Figure 1:**
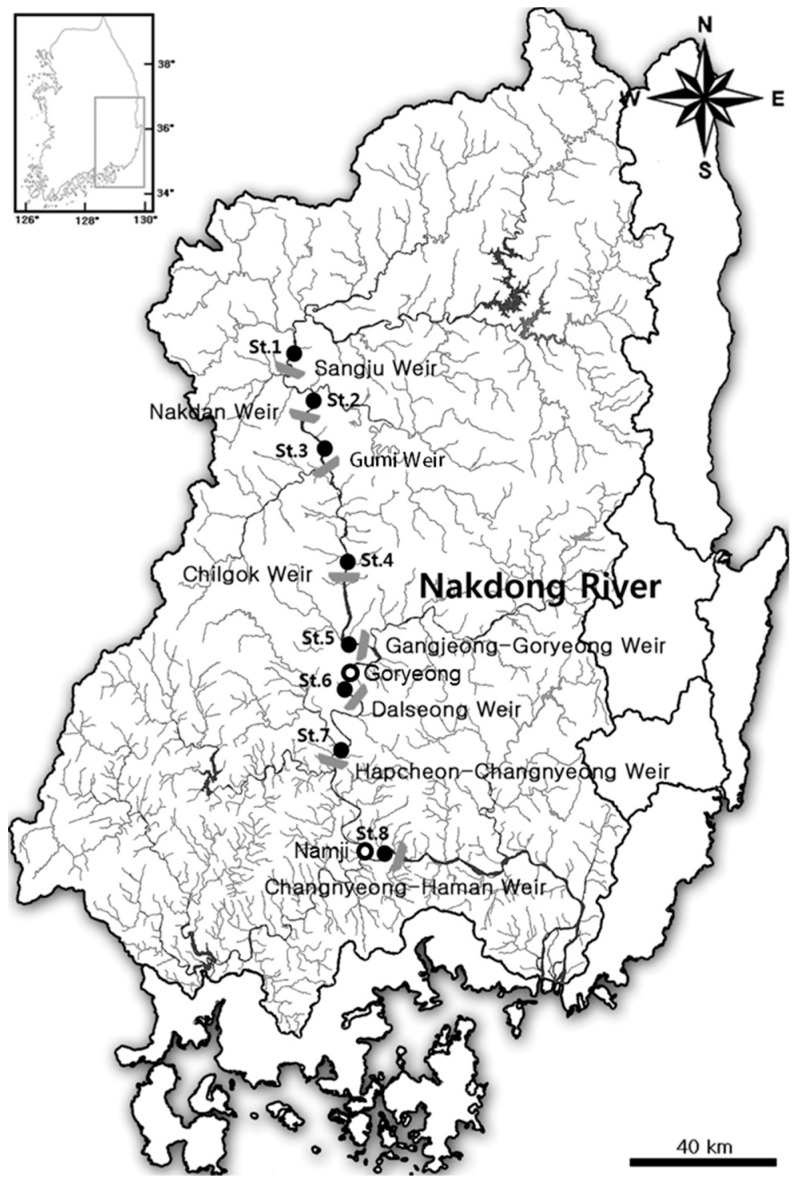
Sampling sites along the Nakdong River, Korea. Gray symbols indicate the major weirs constructed on the mainstream of the river; open and solid circles indicate the monitoring sites along the mainstream before (2006–2007) and after the weir construction (2013), respectively.

The Nakdong River, which originates from Hwangji Pond in Mount Taebaek, is the longest river in South Korea, with its length equal to 510 km. The river crosses the middle-eastern part of South Korea, covering a watershed area of 23,384 km^2^, and flows to the south through the Nakdong Estuary. The Nakdong River has been an important source of drinking, industrial, and agricultural water in the watershed area along the river. Since August 2012, a total of eight weirs (Sangju, Nakdan, Gumi, Chilgok, Gangjeong-Goryeong, Dalseong, Hapcheon-Changnyeong, and Changnyeong-Haman) have been constructed over a stretch of approximately 200 km (Table 1 and Figure 1). The water level between these weirs is maintained by controlling the weir gates. To assess the water quality variables and phytoplankton composition, surface water samples were collected 500 m upstream of each of the eight weirs once a week from January to December 2013. The temperature, pH, dissolved oxygen, and electrical conductivity of the water were measured in situ using a portable water quality meter (YSI 556 MPS; YSI, Yellow Springs, OH, USA) at each sampling site. To analyze chemical quality parameters, water samples were collected in clean polyethylene bottles and immediately transported to the laboratory on ice. To analyze the phytoplankton density, surface water samples were collected from the same sites and preserved by adding 0.3% Lugol’s solution. To compare water quality parameters at all stations (St.), from Sangju (St. 1) to Changnyeong-Haman (St. 8), before and after the construction of the weirs, previous data (2006–2007) on organic matter, including biochemical oxygen demand (BOD_5_) and chemical oxygen demand (COD_Mn_), nutrients, including total nitrogen (TN), nitrate nitrogen (NO_3_^−^-N), ammonia nitrogen (NH_4_^+^-N), total phosphorus (TP), and phosphate phosphorus (PO_4_^3−^-P), and chlorophyll *a* (Chl-*a*) were obtained from the Water Information System (http://water.nier.go.kr) of the Korean Government. Phytoplankton monitoring data from 2006 to 2007 [22] were used to compare the temporal distribution of phytoplankton in the area of the Dalseong weir (located in the middle of the Nakdong River) with that in the areas of the Goryeong and Changnyeong-Haman weirs (located downstream of Namji) (Figure 1). Monthly air temperature and rainfall data, provided by the regional observation stations at Sangju, Gumi, Daegu, Hapcheon, and Milyang, which are the nearest stations to the eight weirs on the Nakdong River, were obtained from the Korea Meteorological Administration website (http://web.kma.go.kr).

### 2.2. Physical and Chemical Variables and Phytoplankton Density

HRT was calculated using discharge rates and the volume provided by K-water (http://kwater.or.kr). BOD_5_ was measured by standard methods [23], and COD_Mn_ was analyzed by estimating the consumption of potassium permanganate. Chl-*a* was analyzed using a spectrophotometer (Lambda 45; PerkinElmer, Waltham, MA, USA), for which the water samples were filtered through Whatman GF-F filters and extracted with 90% acetone for 24 h [23]. TN and TP contents were analyzed using an autoanalyzer (INTEGRAL Futura; Ams Alliance, Frépillon, France). The NO_3_^−^-N content was analyzed using ion chromatography (850 Professional IC; Metrohm, Switzerland). Concentrations of NH_4_^+^-N and PO_4_^3−^-P were determined with an ion analyzer (Smartchem 140; Ams Alliance, Frépillon, France). Phytoplankton samples were identified to the genus or species level following John et al. [24] and Komárek and Anagnostidis [25,26]. For quantitative analysis, 1 mL of a sample was added to and allowed to settle in a Sedgwick–Rafter counting chamber, followed by examination under an optical microscope (Imager M1; Carl Zeiss, Oberkochen, Germany) at a 200× to 400× magnification to calculate the cell density per milliliter.

### 2.3. Data Analysis

Environmental variables were analyzed by the paired *t*-test using the SPSS 12.0 software (SPSS Inc., Chicago, IL, USA) to determine significance of differences between values recorded before (monthly mean data from January to December 2006 and 2007) and after (January to December 2013) the weir construction. A *p*-value of <0.05 was considered significant in all comparisons. Redundancy analysis (RDA) ordination was performed using the CANOCO software version 5.0 [27] to elucidate the relationships between environmental variables and dominant phytoplankton species after the weir construction at representative sites between the Sangju and Changnyeong-Haman weirs.

## 3. Results

### 3.1. Atmospheric, Hydraulic, and Hydrological Conditions in the Weir Section

**Figure 2:**
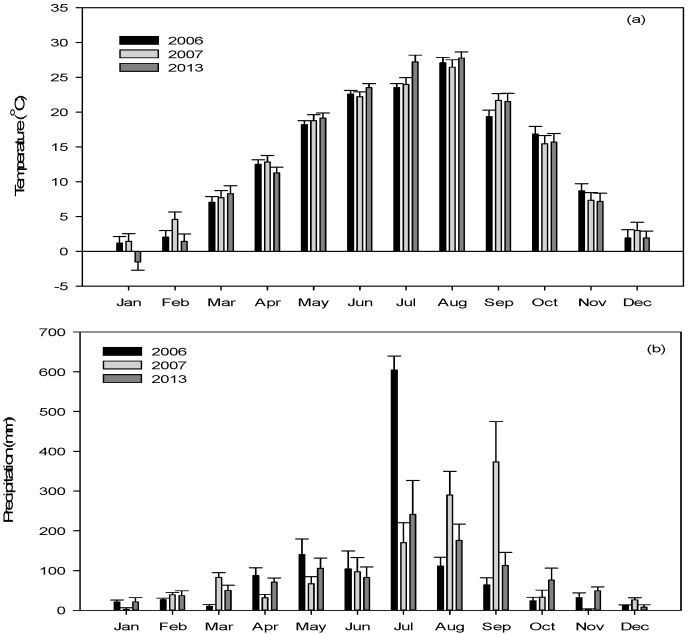
Monthly trends for the air temperature (**a**) and precipitation (**b**) during the study periods before (2006–2007) and after (2013) the weir construction on the Nakdong River.

The mean monthly air temperatures in the Nakdong River weir section ranged from −1.5 to 27.8 °C, and the average annual air temperature was 13.6 °C during the study period from January to December 2013 (Figure 2a). The annual precipitation from January to December 2013 was 1031 mm, thus showing a 16% decrease compared with the mean data obtained during the same period from 2006 to 2007 (1227 mm). The precipitation in July 2013 (241 mm) was the highest monthly record (Figure 2b).

**Figure 3:**
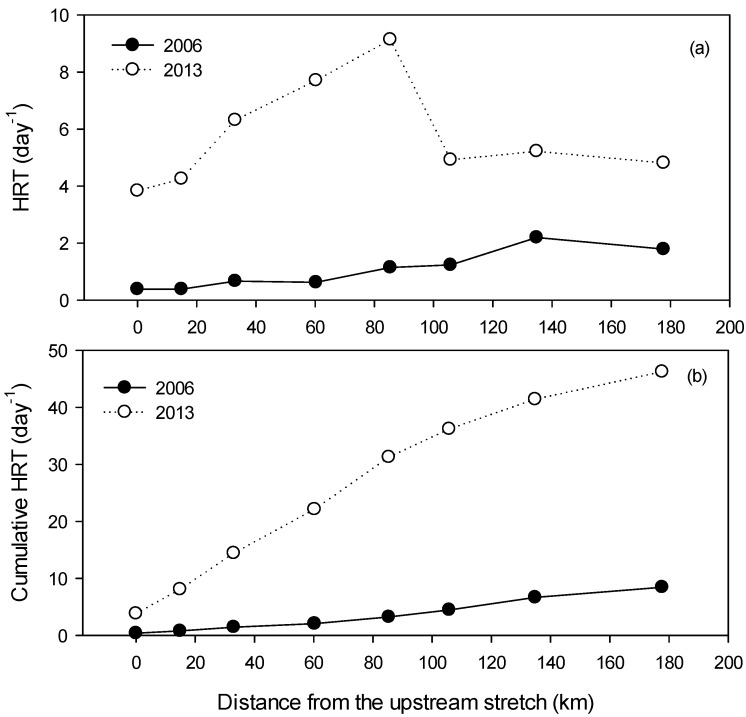
Annual hydraulic retention times (HRTs) in the Nakdong River after the weir construction. (**a**) HRT at each weir section; (**b**) cumulative HRT.

The investigation of hydraulic and hydrological characteristics at the study sites during the study periods after the weir construction revealed that HRT in the Nakdong River was regulated by precipitation and the amount of outflow through regulation of weir gates. The HRT values in the Nakdong River increased 2- to 12-fold (Figure 3a) after the construction of the Hapcheon-Changnyeong and Chilgok weirs respectively, compared to the HRTs of before the weir construction in 2006 [28]. Furthermore, the cumulative HRT between St. 1, located upstream, and St. 8, located downstream, was 46.2 days after the weir construction, i.e., five times longer than that before the construction (Figure 3b).

### 3.2. Variations in Physical and Chemical Parameters

During 2006–2007, before the construction of the eight weirs, the mean BOD_5_ concentrations ranged between 0.8 and 2.8 mg·L^−1^ at the study sites along the Nakdong River. The annual mean BOD_5_ concentration for the upstream stretch, from St. 1 to St. 4, was 1.0 mg·L^−1^. In the middle to lower sections of the Nakdong River, which are represented by the stretch between St. 5 and St. 8, the annual mean BOD_5_ concentration was 2.6 mg·L^−1^, indicating that organic pollution increased from the upstream to downstream areas. In 2013, after the weir construction, the annual mean BOD_5_ concentrations ranged from 1.9 to 2.5 mg·L^−1^ at the weir sites. In the upstream stretch, from St. 1 to St. 4, the average value was 2.3 mg·L^−1^, which indicated that organic pollution levels increased after the weir construction (*p* < 0.05). By contrast, in the middle to lower parts of the river, from St. 5 to St. 8, the annual mean BOD_5_ concentration was 2.4 mg·L^−1^, which was lower than that before the construction, but there were no statistically significant differences (*p* > 0.05). These results showed that the organic pollution levels were different in each stretch of the Nakdong River after the weir construction (Table 2).

The annual mean COD_Mn_ concentration in the Nakdong River during 2006–2007, before the weir construction, was 4.6 mg·L^−1^; the values were 3.5 mg·L^−1^ in the upstream stretch, from St. 1 to St. 4, and 5.8 mg·L^−1^ in the middle to lower stretch, between St. 5 and St. 8. In 2013, after the weir construction, the COD_Mn_ concentration increased in the upstream stretch between St. 1 and St. 4 (*p* < 0.01) but did not significantly change in the downstream stretch (Table 2). The annual mean TP concentrations ranged between 0.047 and 0.200 mg·L^−1^ at the eight sites during 2006–2007 and were usually high in the middle and downstream stretch of the Nakdong River, except St. 5 (Table 2). However, after the weir construction, the annual TP concentrations ranged between 0.044 and 0.075 mg·L^−1^ at the weir sites. The middle to lower sections of the Nakdong River, represented by the stretch between St. 4 and St. 8, exhibited a significant decrease in TP concentrations (*p* < 0.05) (Table 2). The annual mean TN concentrations at the eight sites ranged between 2.457 and 4.045 mg·L^−1^ during 2006–2007. After the weir construction, the TN concentrations at the sites ranged from 2.506 to 3.699 mg·L^−1^, showing spatial differences after the construction of the weirs.

The annual mean Chl-*a* concentrations at the study sites ranged from 7.7 to 58.7 mg·m^−3^ in 2006 and 2007, before the weir construction, and from 18.6 to 32.7 mg·m^−3^ in 2013, after the weir construction. In the upper stretch, including St. 1, St. 2, and St. 3, the concentration of Chl-*a* increased after the weir construction (*p* < 0.05); however, it decreased by approximately 50% in the downstream stretch, at St. 7 and St. 8 (*p* < 0.05), after the weir construction (Table 2).

**Figure 4:**
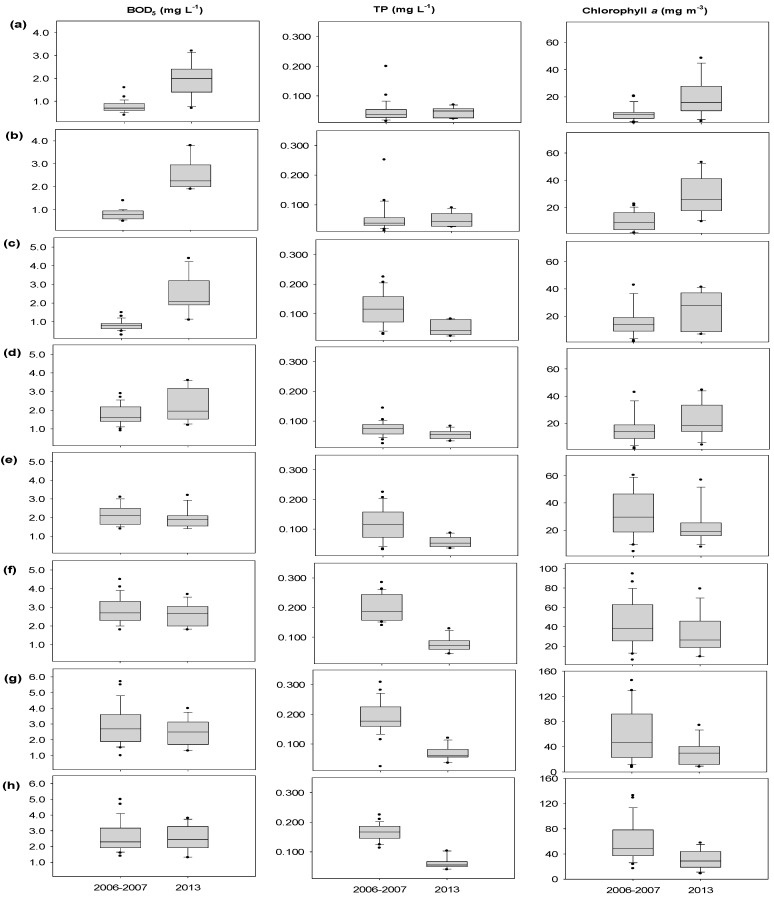
Spatial variations in biochemical oxygen demand (BOD_5_), total phosphorus (TP), and chlorophyll *a* concentration in the Nakdong River before (2006–2007) and after (2013) the weir construction. Box plots indicate the minimum, 10%, 25%, median, 75%, 90%, and maximum levels at (**a**) Sangju weir (St. 1); (**b**) Nakdan weir (St. 2); (**c**) Gumi weir (St. 3); (**d**) Chilgok weir (St. 4); (**e**) Gangjeong-Goryeong weir (St. 5); (**f**), Dalseong weir (St. 6); (**g**) Hapcheon-Changnyeong weir (St. 7); and (**h**) Changnyeong-Haman weir (St. 8).

The box plots of BOD_5_, TP, and Chl-*a* concentrations, shown in Figure 4, present differences in the water quality variables at the eight study sites, detected by paired *t*-test analysis. Comparison of the data obtained before and after the weir construction showed significant increases in the mean BOD_5_ (*p* < 0.001) and Chl-*a* concentrations (*p* < 0.05) in the upper stretch, St. 1, St. 2, and St. 3 (Figure 4a–c). Considerable spatial and temporal differences were detected in the TP concentrations from St. 4 to St. 8. after the construction of the eight weirs (*p* < 0.05), (Figure 4d–h). Lower Chl-*a* concentrations were also observed at the downstream weir sites, St. 7 and St. 8, in 2013 (*p* < 0.05).

### 3.3. Variations in Phytoplankton Density

**Figure 5:**
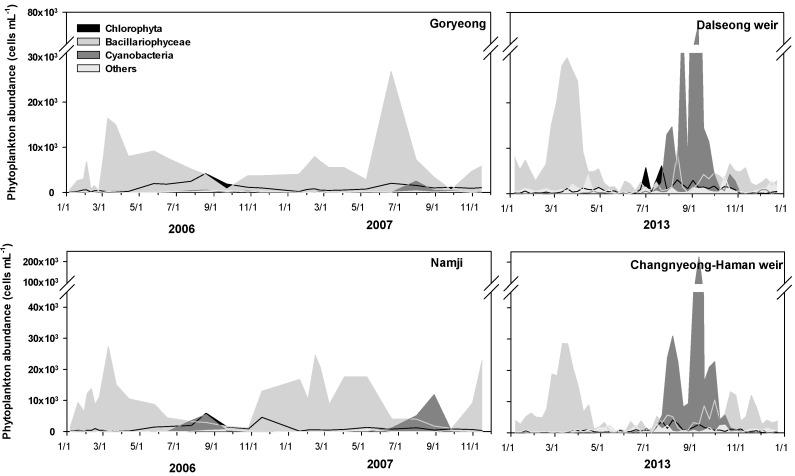
Comparison of the phytoplankton cell density before (2006–2007) and after (2013) the construction of the Goryeong, Dalseong, Namji, and Changnyeong-Haman weirs.

During 2006–2007, before the weir construction, diatoms dominated the study sites, including Goryeong and Namji in the middle and downstream sections of the Nakdong River, in winter and spring. Small numbers of green algae and cyanobacteria occasionally appeared during this period. The dominant species in the Nakdong River were the centric diatom *Stephanodiscus hantzschii* and the colonial cyanobacterium *Microcystis aeruginosa* from winter to the following spring and from summer to fall, respectively (Figure 5).

**Figure 6:**
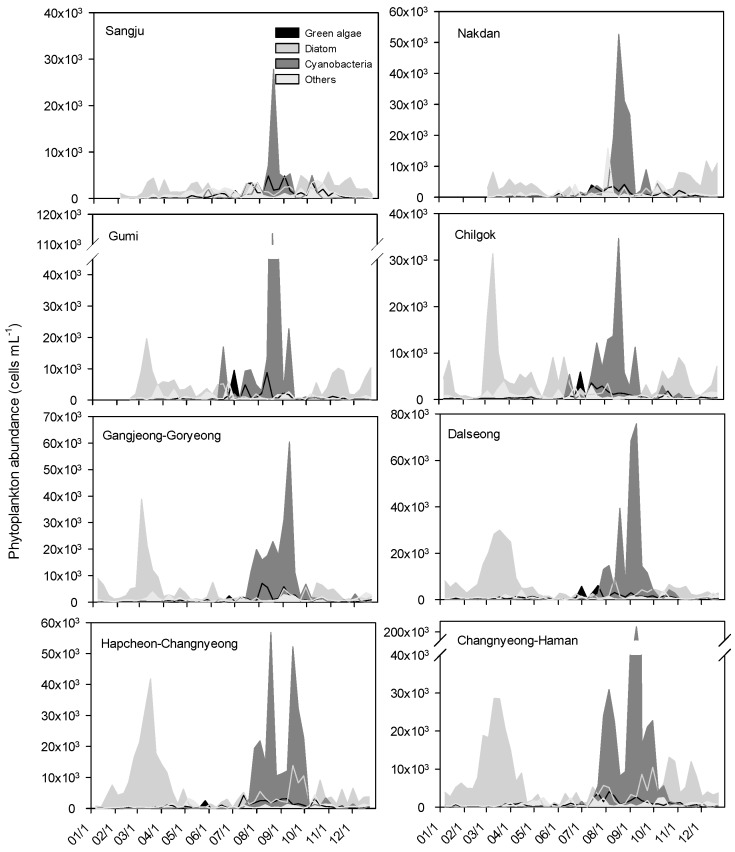
Temporal and spatial changes in the phytoplankton cell density in the Nakdong River after construction of the eight weirs (2013).

In 2013, after the weir construction, the diatom *S. hantzschii* also dominated from fall to the following spring (November to April); however, the cyanobacterium *M. aeruginosa* proliferated to a greater extent during summer and fall (June to September). The phytoplankton cell densities ranged from 8.4 × 10^3^ to 17.5 × 10^3^ cells·mL^−1^ in the upstream stretch, between the Sangju and Chilgok weirs, and from 10.2 × 10^3^ to 15.8 × 10^3^ cells·mL^−1^ in the midstream stretch, from the Gangjeong-Goryeong to Hapcheon-Changnyeong weirs; the density was 19.6 × 10^3^ cells·mL^−1^ at the downstream Changnyeong-Haman weir (Figure 6).

### 3.4. Correlation between Phytoplankton Species and Environmental Variables

**Figure 7:**
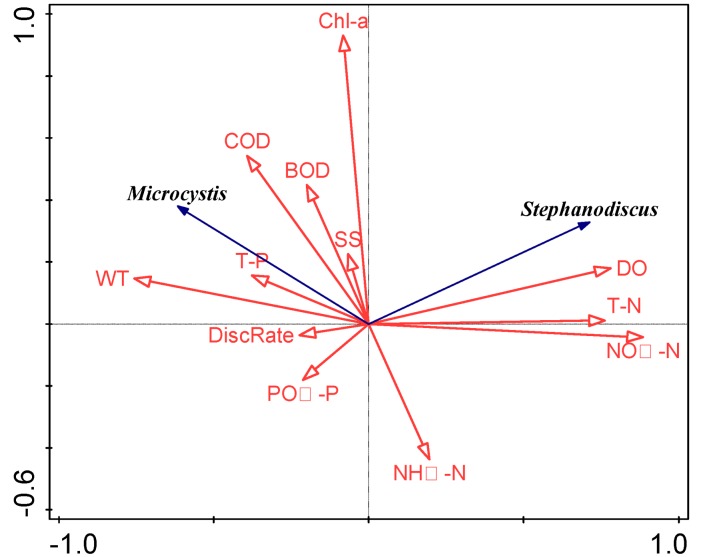
Ordination biplots of environmental variables and dominant species, obtained using redundancy analysis, in the Nakdong River weir section. Variables included the discharge rate (DiscRate), water temperature (WT), dissolved oxygen (DO), biochemical oxygen demand (BOD_5_), chemical oxygen demand (COD_Mn_), suspended solids (SS), chlorophyll *a* (Chl-*a*), total nitrogen (TN), total phosphorus (TP), nitrate nitrogen (NO_3_^−^-N), ammonia nitrogen (NH_4_^+^-N), and phosphate phosphorus (PO_4_^3−^-P).

The correlation between environmental variables and major phytoplankton species after the weir construction was determined by RDA (Figure 7). The first two axes explained 57.0% of the total variance, showing a positive correlation between *Microcystis* and the water temperature, TP, COD_Mn_, BOD_5_, and Chl-*a*, while *Stephanodiscus* negatively correlated with the water temperature, but was closely related to DO and TN.

## 4. Discussion

In recent years, eight multifunctional weirs have been constructed along the Nakdong River section stretching for approximately 200 km to (1) ensure sufficient flow during the dry season for agricultural uses; (2) maintain environmental flow to the river stretch; and (3) enhance the water quality in the river. The physical environment, such as the water depth, flow rate, and velocity, has changed in the Nakdong River weir section [29,30,31]. The HRT values in the upper stretch (St. 1 to St. 4) ranged between 2.7 and 7.3 days, showing a 3- to 10-fold increase, while the HRT values in the lower part of the river (St. 5 to St. 8) ranged between 3.1 and 8.4 days, indicating a 2- to 6-fold increase compared with those before the weir construction, based on the flow travel time simulation [30].

In the present study, the physical and chemical environments varied between the upstream and downstream stretches of the Nakdong River. The BOD_5_ concentrations increased at the upper three sites, from St. 1 to St. 3, while only marginal changes were observed in the downstream stretch, including St. 4, after the weir construction. The BOD_5_ concentration in the upper stretch was affected by changes in the phytoplankton cell density, based on the Chl-*a* concentrations before and after the weir construction. Wang et al. [32] found that the BOD_5_ concentration significantly positively correlates with the phytoplankton density during spring, summer, and autumn. In the present study, at three sites between the St. 1 and St. 3, the TP concentration exhibited marginal variations; however, the average Chl-*a* concentration increased approximately two-fold in 2013 compared with that before the weir construction. Wehr and Descy [33] reported that the levels of nutrients in rivers are often higher than algal requirements. Therefore, the phytoplankton cell density and production are often controlled by the discharge rate, which is related to the residence time, dilution rate, and other factors.

In the present study, in the stretch between St. 4 and St. 8, which corresponds to the middle and lower parts of the Nakdong River, the TP concentration decreased sharply, except at St. 5, compared with that before the weir construction. The Chl-*a* concentration also decreased compared with that before the weir construction. Based on the reduced nutrient and Chl-*a* concentrations, changes in the physical and chemical environments of the water in the middle and downstream stretches of the Nakdong River may be attributed to a higher dilution factor with the increase in the water depth and volume [30]. Furthermore, the loss of phytoplankton from sedimentation would also change because of a longer HRT, and the P input might be reduced by managing the wastewater treatment plant effluents, as well as industrial and municipal discharges, after the weir construction. [34].

There was no significant change in the seasonal succession of dominant species before (2006–2007) and after the weir construction (2013). However, the cell density of *Stephanodiscus hantzschii*, which usually dominated from fall to the following spring, was 6.0 × 10^3^ cells·mL^−1^ after the weir construction, showing a 20% decline compared with that before the weir construction

According to previous studies, the diatom growth in spring mainly depends on the water temperature, nutrient availability (silica and P), light intensity, and carbon availability [35,36,37]. Diatom growth in spring may be primarily attributed to the high growth rate of *S. hantzschii* at low water temperatures and to its superior competitive ability over other diatoms during the period of low nutrient concentrations as small centric diatoms have a half-saturation growth constant of <10 µg·L^−1^ for P [34,38]. Salmaso and Braioni [39] have also reported that a low flow rate is essential for the growth of planktonic algae, including small centric diatoms, in river systems, and low flow rates and water mixing during dry seasons can lead to downstream accumulation of diatom cells in large river systems. In the present study, the centric diatom cell density declined after the weir construction. This may be attributed to a complex combined effect of physical, chemical, and biological factors. Sedimentation can be one of the selective factors that suppresses the growth of centric diatoms and prevent their dominance in shallow river stretches [14]. Köhler [40] has suggested that centric diatoms would decline in lakes because the zooplankton density increase in spring, and there would be a shift toward growth of buoyant cyanobacteria. Moreover, the reduced flow with an increased HRT might have enhanced the grazing pressure on small centric diatoms in the main river system after the construction of the weirs. However, zooplankton density was not measured in the present study.

One of the most notable differences in the phytoplankton distribution between the periods before and after the weir construction was an increase in the frequency, intensity, and duration of cyanobacterial blooms. Among cyanobacterial species, *Microcystis aeruginosa*, which is generally dominant during summer and fall, increased more than 10-fold, from an average of 0.5 × 10^3^ cells·mL^−1^ before the weir construction to an average of 7.0 × 10^3^ cells·mL^−1^ after the construction. Hilton et al. [41] have demonstrated that large, deep, and impounded rivers are usually characterized by long retention times, which are greater than algal doubling times. Therefore, a high biomass of phytoplankton can accumulate in the middle and lower stretches of a river. Furthermore, Reynolds [14] has suggested that the lower end of the range of retention times, covering the transition range, is likely to be between 4 and 6 days, which, based on the doubling time of common cyanobacteria, would correspond to 0.3 to 1.4 doublings per day. The HRT values in the eight weir sections ranged between 2 and 8 days during the bloom period of *M. aeruginosa* in 2013. Thus, both the high-water temperature (>20 °C) and retention time, as well as sufficient nutrients such as TP, might have led to the increased growth of cyanobacteria in the Nakdong River after the weir construction. These results were similar to the studies that the *Microcystis* growth was positively correlated with phosphorus concentration when it was lower than 0.445 mg L^−1^ [42]. These findings suggest that meteorological parameters, such as the temperature and precipitation, may affect the time of the occurrence of cyanobacterial blooms in the weir sections. Thus, appropriate regulation of water discharges in the weir stretches, based on the weather conditions, may directly affect the occurrence of blooms and the composition of phytoplankton in the weir system.

## 5. Conclusions

To the best of our knowledge, the present study is the first to demonstrate changes in limnological characteristics, including physical, chemical, and biological parameters, after consecutive construction of weirs in a large river system. The results revealed spatial variations in the TP and Chl-*a* concentrations among consecutive weir sections. The phytoplankton community composition also changed after the weir construction. The average cell density of *Stephanodiscus hantzschii* marginally decreased after the construction; however, proliferation of *Microcystis aeruginosa* was more intense during the summer and fall seasons, as compared with that before the weir construction. Therefore, appropriate regulation of the water discharge in weir stretches, based on environmental factors, can be implemented to reduce the occurrence of cyanobacterial blooms in the weir system.

## Figures and Tables

**Table 1:** Physical parameters of the study sites.

Site	Elevation (EL m)	Distance (km)	Volume (10^6^ m^3^)	Catchment Area (km^2^)	Average Depth (m)	Geographic Coordinates (Latitude/Longitude)
Sangju (St. 1)	47.0	28.2	27	7407	11.0	36°25′51.1″ N/128°15′5.19″ E
Nakdan (St. 2)	40.0	15.7	35	9221	11.5	36°21′28.16″ N/128°18′27.96″ E
Gumi (St. 3)	32.5	18.2	53	9557	12.0	36°14′2.11″ N/128°20′52.48″ E
Chilgok (St. 4)	25.5	27.5	75	11,040	11.5	36°0′45.51″ N/128°24′3.58″ E
Gangjeong-Goryeong (St. 5)	19.5	26.2	92	11,667	10.5	35°50′27.73″ N/128°27′31.1″ E
Dalseong (St. 6)	14.0	19.2	59	14,248	10.5	35°43′55.18″ N/128°25′10.9″ E
Hapcheon-Changnyeong (St. 7)	10.5	29.8	70	15,074	9.0	35°35′16.13″ N/128°21′29.39″ E
Changnyeong-Haman (St. 8)	5.0	42.5	101	20,697	13.2	35°22′39.42″ N/128°33′15.96″ E

**Table 2:** Environmental variables measured in the surface water at the study sites.

Parameter	Year(s)	Sangju (St. 1)	Nakdan (St. 2)	Gumi (St. 3)	Chilgok (St. 4)	Gangjeong-Goryeong (St. 5)	Dalseong (St. 6)	Hapcheon-Changnyeong (St. 7)	Changnyeong-Haman (St. 8)
Temperature (°C)	2006–2007 2013	15.5 ± 8.4 17.4 ± 9.8	15.2 ± 8.7 19.1 ± 8.7 *	15.5 ± 8.9 18.5 ± 10.0 *	15.4 ± 8.0 17.1 ± 110.5	16.2 ± 8.9 16.4 ± 10.0	16.3 ± 8.3 17.1 ± 9.8	16.5 ± 8.7 17.5 ± 9.9	16.4 ± 8.5 17.9 ± 10.3
pH	2006–2007 2013	7.9 ± 0.2 8.4 ± 0.4 *	7.8 ± 0.2 8.6 ± 0.2 ***	7.9 ± 0.2 8.6 ± 0.4 ***	7.8 ± 0.3 8.4 ± 0.4 ***	8.2 ± 0.4 8.2 ± 0.3	8.0 ± 0.4 8.2 ± 0.3 *	7.7 ± 0.7 8.3 ± 0.3 **	7.6 ± 0.6 8.4 ± 0.3 **
DO (mg·L^−1^)	2006–2007 2013	9.2 ± 2.1 11.5 ± 1.3 *	8.9 ± 2.1 11.1 ± 1.7 ***	9.1 ± 2.1 11.2 ± 1.9 ***	9.1 ± 2.1 11.3 ± 2.3 ***	11.4 ± 2.1 11.2 ± 2.6	10.7 ± 2.2 11.5 ± 2.6 *	11.0 ± 3.1 11.5 ± 2.6	10.9 ± 2.7 11.4 ± 2.2
Conductivity (µS·cm^−1^)	2006–2007 2013	125 ± 25 189 ± 27 ***	128 ± 22 206 ± 23 ***	129 ± 25 195 ± 24 ***	202 ± 50 244 ± 42 *	250 ± 48 244 ± 40	383 ± 107 349 ± 82	305 ± 58 324 ± 71	255 ± 37 268 ± 50
BOD_5_ (mg·L^−1^)	2006–2007 2013	0.8 ± 0.2 1.9 ± 0.7 ***	0.8 ± 0.2 2.5 ± 0.7 ***	0.8 ± 0.2 2.4 ± 1.0 ***	1.8 ± 0.4 2.3 ± 0.9	2.2 ± 0.5 1.9 ± 0.5	2.8 ± 0.7 2.6 ± 0.6	2.9 ± 1.0 2.5 ± 0.8	2.6 ± 0.9 2.6 ± 0.8
COD_Mn_ (mg·L^−1^)	2006–2007 2013	3.1 ± 0.9 4.3 ± 0.8 **	3.2 ± 1.0 5.3 ± 1.0 ***	3.3 ± 0.9 5.3 ± 1.2 ***	4.3 ± 0.9 5.5 ± 1.2 **	4.9 ± 0.7 5.4 ± 0.9	6.2 ± 0.9 6.6 ± 1.0	6.3 ± 1.0 6.4 ± 1.2	5.9 ± 1.0 6.2 ± 1.2
Chlorophyll *a* (mg·m^−3^)	2006–2007 2013	7.7 ± 3.7 18.6 ± 12.7 *	9.9 ± 5.6 29.3 ± 14.1 *	11.5 ± 7.1 24.6 ± 13.0 **	17.2 ± 13.4 23.2 ± 12.8	33.6 ± 18.4 22.9 ± 13.2	44.2 ± 23.1 32.7 ± 19.8	57.0 ± 36.0 29.6 ± 19.3 *	58.7 ± 29.4 31.2 ± 14.7 *
TN (mg·L^−1^)	2006–2007 2013	2.457 ± 0.288 2.506 ± 0.331	2.509 ± 0.336 2.470 ± 0.315	2.478 ± 0.344 2.465 ± 0.391	3.162 ± 0.610 2.869 ± 0.494 *	2.830 ± 0.477 2.718 ± 0.542	4.045 ± 0.926 3.699 ± 0.829 **	3.669 ± 0.863 3.343 ± 0.718 *	3.249 ± 0.667 2.944 ± 0.641 **
NH_3_^+^-N (mg·L^−1^)	2006–2007 2013	0.069 ± 0.050 0.049 ± 0.050	0.061 ± 0.036 0.040 ± 0.019 **	0.052 ± 0.027 0.053 ± 0.040	0.290 ± 0.268 0.099 ± 0.066 **	0.115 ± 0.099 0.083 ± 0.044	0.194 ± 0.147 0.108 ± 0.052 *	0.088 ± 0.071 0.090 ± 0.051	0.064 ± 0.062 0.072 ± 0.041
NO_3_^−^-N (mg·L^−1^)	2006–2007 2013	2.172 ± 0.360 2.011 ± 0.343	2.272 ± 0.336 1.839 ± 0.420	2.263 ± 0.344 1.841 ± 0.411 **	2.551 ± 0.426 2.101 ± 0.480 ***	2.319 ± 0.038 1.960 ± 0.502 ***	3.199 ± 0.813 2.722 ± 0.775 ***	2.388 ± 0.604 2.456 ± 0.704	2.014 ± 0.439 2.095 ± 0.062
TP (mg·L^−1^)	2006–2007 2013	0.047 ± 0.026 0.044 ± 0.017	0.053 ± 0.038 0.050 ± 0.023	0.054 ± 0.038 0.051 ± 0.025	0.116 ± 0.047 0.057 ± 0.018 **	0.074 ± 0.017 0.054 ± 0.015 *	0.200 ± 0.037 0.075 ± 0.025 ***	0.189 ± 0.037 0.069 ± 0.024 ***	0.165 ± 0.021 0.063 ± 0.020 ***
PO_4_^3−^-P (mg·L^−1^)	2006–2007 2013	0.018 ± 0.010 0.009 ± 0.009	0.019 ± 0.012 0.006 ± 0.007 **	0.016 ± 0.013 0.008 ± 0.008 *	0.070 ± 0.037 0.012 ± 0.009 ***	0.016 ± 0.010 0.010 ± 0.006	0.121 ± 0.041 0.013 ± 0.011 ***	0.097 ± 0.023 0.013 ± 0.011 ***	0.071 ± 0.011 0.008 ± 0.008 ***

Notes: Values are the mean ± standard deviation. DO, dissolved oxygen; BOD_5_, biological oxygen demand; COD_Mn_, chemical oxygen demand; TN, total nitrogen; NH_4_^+^-N, ammonia nitrogen; NO_3_^−^-N, nitrate nitrogen; TP, total phosphorus; PO_4_^3−^-P, phosphate phosphorus. Statistically significant differences are indicated: *** *p* ≤ 0.001; ** *p* < 0.01; * *p* < 0.05.
